# Snake venom disintegrins update: insights about new findings

**DOI:** 10.1590/1678-9199-JVATITD-2023-0039

**Published:** 2023-09-18

**Authors:** Gabriela de Oliveira Almeida, Isadora Sousa de Oliveira, Eliane Candiani Arantes, Suely Vilela Sampaio

**Affiliations:** 1 Department of Clinical Analysis, Toxicology and Food Science, School of Pharmaceutical Sciences of Ribeirão Preto, University of São Paulo, Ribeirão Preto, SP, Brazil.; 2 Department of BioMolecular Sciences, School of Pharmaceutical Sciences of Ribeirão Preto, University of São Paulo, Ribeirão Preto, SP, Brazil.; 3 Department of Biotechnology and Biomedicine, Technical University of Denmark, Kongens Lyngby, Denmark.

**Keywords:** disintegrins, SVMP, ADAM, snake venom, integrins, RGD domain

## Abstract

Snake venom disintegrins are low molecular weight, non-enzymatic proteins rich in cysteine, present in the venom of snakes from the families Viperidae, Crotalidae, Atractaspididae, Elapidae, and Colubridae. This family of proteins originated in venom through the proteolytic processing of metalloproteinases (SVMPs), which, in turn, evolved from a gene encoding an A Disintegrin And Metalloprotease (ADAM) molecule. Disintegrins have a recognition motif for integrins in their structure, allowing interaction with these transmembrane adhesion receptors and preventing their binding to proteins in the extracellular matrix and other cells. This interaction gives disintegrins their wide range of biological functions, including inhibition of platelet aggregation and antitumor activity. As a result, many studies have been conducted in an attempt to use these natural compounds as a basis for developing therapies for the treatment of various diseases. Furthermore, the FDA has approved Tirofiban and Eptifibatide as antiplatelet compounds, and they are synthesized from the structure of echistatin and barbourin, respectively. In this review, we discuss some of the main functional and structural characteristics of this class of proteins and their potential for therapeutic use.

## Background

Snake venom is a secretion produced in the glands located on both sides of the animal's upper jaw. Its evolutionary function includes the defense and survival of the snake, as well as the immobilization and digestion of prey, aiding in its feeding. It is a complex cocktail, as its composition is formed by the mixture of various compounds, predominantly proteins, peptides, amino acids, nucleic acids, carbohydrates, lipids, and metals [1, 2]. After its production in pairs of homologous glands, venom is secreted into the base of the fangs, which can be located in the posterior region (opisthoglyphous) or anterior region of the animal's mouth, with the latter case having either short and fixed fangs (proteroglyphous) or long and movable fangs (solenoglyphous) [2, 3].

Snakebite envenomation is considered a Neglected Tropical Disease with high incidence and severity, mainly affecting poverty regions [4]. It is estimated that around 5.4 million snakebites occur worldwide each year, resulting in 1.8 to 2.7 million cases of envenomation and approximately 81,000 to 138,000 deaths [5]. Snake venom exhibits a highly complex composition, and due to the diverse toxins with a wide range of biological functions, various clinical manifestations resulting from envenomation are observed, including local and systemic effects [6]. However, beyond its toxic action, snake venom is also recognized for its high therapeutic potential, as its composition contains approximately 100 to 500 pharmacologically active compounds capable of acting on different target sites. For this reason, many studies have been conducted in the search for alternative therapies for various diseases [7].

In this context, snake venomics has demonstrated great relevance for the more detailed analysis of venom components [8]. By using this strategy, which combines advances in proteomics and transcriptomics, it is possible to isolate venom compounds, estimate the content of toxins, as well as understand their biological and toxicological aspects [9]. Advances in these techniques have allowed the characterization of up to 20 families of proteins in the venom of a single snake, with some of these families containing up to 80 different toxins [10]. Despite the fascinating variability of compounds, most snake venoms are composed of four dominant protein families: phospholipase A_2_ (PLA_2_), three-finger toxins (3FTx), snake venom serine protease (SVSP), and snake venom metalloprotease (SVMP), along with secondary protein families, such as cysteine-rich secretory protein (CRISP), Kunitz peptides, L-amino acid oxidase (LAAO), natriuretic peptides, C-type lectins (CTL), disintegrins, among others [11].

In this review, we present the functional and structural aspects of disintegrins found in snake venom, as well as the evolutionary history of their emergence. We also discuss the potential applications of this class of peptides and the drugs already approved for therapeutic use.

### What are snake venom disintegrins?

Snake venom disintegrins comprise a family of highly homologous, non-enzymatic polypeptides rich in cysteine (Cys). Their presence is described in the venom of snakes from the families Viperidae, Crotalidae, Atractaspididae, Elapidae, and Colubridae [12]. This family of small proteins interacts specifically with integrins, a group of cell adhesion receptors on the surface of certain cells, including platelets, vascular endothelial cells, and some tumor cells [13, 14]. This way, disintegrins, by preventing such binding, interfere in intercellular and cell-matrix interactions, as well as signal transduction [12, 14]. 

### Integrins: a family of heterodimeric receptors

Integrins are transmembrane receptors that regulate or trigger different cellular processes upon binding to specific extracellular ligands [15]. They are heterodimeric proteins formed by the non-covalent association of α and β chains. In vertebrates, at least 18 α subunits and 8 β subunits have been identified, which can form a total of 24 different heterodimers. The α and β subunits of integrins do not have detectable homology between them, but there are conserved regions among different α subunits (approximately 30% identity) and among β subunits (around 45%) [16]. 

**Figure 1.**
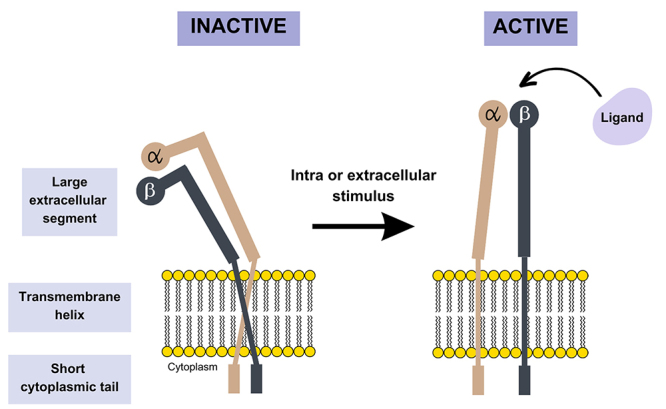
Integrin structure. Conversion of integrin from its inactive low-affinity conformation to the active high-affinity conformation for the ligand through intra- or extracellular stimuli.

Integrins can recognize ligands from the extracellular matrix, cell surfaces, and other soluble ligands, with the αβ pairings of integrin subunits being determinants for binding specificity [16, 17]. Structurally, each integrin subunit consists of an extended multidomain extracellular region (up to 1104 residues in the α subunit and 778 residues in the β subunit), a transmembrane helix, and a short cytoplasmic tail (with 20 to 70 amino acids). The N-terminal portions of each subunit, located in the extracellular region, combine to form a globular ligand-binding "head" (Figure 1) [18, 19].

Integrins are present on the surface of many cell types and enable cell-cell interactions and interactions between cells and extracellular matrix proteins, including fibronectin, collagen, and laminin-1 [20]. These interactions are related to a wide range of biological effects, so the role of integrins is associated with physiological events such as cell adhesion [21], wound healing [22], regulation of neuronal connectivity [23], and synapses [24], as well as pathological effects as inflammation [17], tissue fibrosis [25], atherosclerotic plaque development [26], They also interfere in various stages of cancer development and progression, including survival, proliferation, angiogenesis, migration, invasion, survival in circulation, extravasation, and metastatic growth [12, 15, 17, 27-31]. 

### Snake venom disintegrins: evolution from metalloproteases

Snake venom disintegrins are peptides derived from the proteolytic processing of snake venom metalloproteinase (SVMP) precursors and carry in their structure the recognition motifs for integrins RGD, KGD, WGD, VGD, MGD, RTS, KTS [13, 32]. SVMPs are found in large quantities in snake venom and are the main components responsible for the hemorrhagic action after snakebite, interfering with the victim's hemostatic system [33, 34]. They are divided into different subclasses based on size and domain structure. Class P-I SVMPs contain only the typical metalloproteinase domain (M), composed of the pro-domain and proteolytic domain, and have a molecular mass of 20 to 30 kDa. Class P-II SVMPs have a molecular mass of 30 to 60 kDa and are structurally composed of pro-domain, proteolytic domain, and disintegrin-like domain (DI). Class P-III SVMPs (hemorrhagins) have a molecular mass between 60 to 100 kDa and are composed of a pro-domain, proteolytic domain, a disintegrin-like domain, and a cysteine-rich domain (C). In general, the hemorrhagic activity of these toxins depends on the M domain, but the DI and C domains are also important for their biological function. Thus, class P-III is recognized for its ability to induce higher and more diverse hemorrhagic activity when compared to class P-I and P-II SVMPs [33, 35, 36].

Evidence from molecular phylogenetics suggests that SVMPs evolved from a gene that encodes an A Disintegrin And Metalloprotease (ADAM) molecule, likely from an ancestral ADAM 7 or ADAM 28, belonging to the adamalysin family. Evolutionarily, SVMPs were recruited to the snake venom gland at the base of the advanced snake radiation, after the divergence of Pareatidae from the remaining Caenophidians, during the Paleogene period of the Cenozoic Era. The evolutionary history of SVMPs shows the loss of the cysteine-rich domain in class P-III, forming the SVMPs-PII, followed by the loss of the disintegrin-like domain and the formation of class P-I [35, 37]. 

Regarding domain organization and sequence, important similarities are observed between ADAMs and P-III SVMPs, including the presence of the pro-domain, proteolytic domain, disintegrin-like domain, and cysteine-rich domain. Regarding structural differences, ADAMs have an EGF domain, a transmembrane domain, and a cytoplasmic tail, which are not present in SVMPs [38]. 

The evolutionary history of disintegrins occurred through positive Darwinian selection, and their presence in snake venom results from the proteolytic processing of P-II metalloproteinases or translation of short messenger RNAs without the metalloproteinase coding region [39-42]. Thus, the presence of both free metalloproteinases and disintegrins can be observed in the venom [43].

### Discovery and distribution of snake venom disintegrins

Snake venom disintegrins emerged in the scientific community in 1987, when Stefan Niewiarowski and Tur-Fu Huang isolated a low molecular weight non-enzymatic protein from the venom of *Trimeresurus gramineus*. The researchers observed that the protein, called trigramin, could block the binding of fibrinogen to stimulated GPIIb/IIIa receptors on platelets, thus inhibiting platelet aggregation. Although introduced in Toxinology in 1987, the term "disintegrin" was first used in 1990 when it was described as a new class of peptides isolated from snake venom, rich in the amino acid cysteine and containing an RGD domain in their structure [44, 45]. Since then, numerous studies have been conducted searching for this class of compounds in snake venom (Table 1). Approximately ten years after its discovery, non-RGD disintegrins were identified, challenging the concept of the obligatory presence of the Arg-Gly-Asp amino acids, and paving the way for the future discovery of different integrin recognition motifs [46, 47].

**Table 1.** Snake venom disintegrins isolation.

Disintegrin	Snake venom species	Motif	Publication data	Ref.
Trigramin	*Trimeresurus gramineus*	RGD	November-87	[44]
Echistatin	*Echis carinatus*	RGD	December-88	[76]
Applaggin	*Agkistrodon piscivorus piscivorus*	RGD	October-89	[110]
Albolabrin	*Trimeserusus albolabris*	RGD	May-90	[111]
Elegantin	*Trimeserusus elegans*	RGD	May-90	[111]
Flavoridin	*Trimeserusus flavoviridis*	RGD	July-90	[112]
Batroxostatin	*Bothrops atrox*	RGD	September-90	[81]
Eristostatin	*Eristicophis macmahoni*	RGD	November-90	[45]
Rhodostomin	*Calloselasma rhodostoma*	RGD	November-90	[45]
Triflavin	*Protobothrops flavoviridis*	RGD	February-91	[113]
Barbourin	*Sistrurus miliarius barbouri*	KGD	May-91	[78]
Basilicin	*Crotalus basilicus*	RGD	January-93	[84]
Cerastin	*Cerastes cereastes*	RGD	January-93	[84]
Cereberin	*Crotalus viridis cereberus*	RGD	January-93	[84]
Crotatoxin	*Crotalus atrox*	RGD	January-93	[84]
Cotiarin	*Bothrops cotiara*	RGD	January-93	[84]
Durissin	*Crotalus durissus durissus*	RGD	January-93	[84]
Jararacin	*Bothrops jararaca*	RGD	January-93	[84]
Lachesin	*Lachesis mutus*	RGD	January-93	[84]
Lutosin	*Crotalus viridis lutosus*	RGD	January-93	[84]
Molossin	*Crotalus molossus molossus*	RGD	January-93	[84]
Viridin	*Crotalus viridis viridis*	RGD	January-93	[84]
Contortrostatin	*Agkistrodon contortrix contortrix*	RGD	January-94	[114]
Multisquamatin	*Echis multisquamatus*	RGD	January-94	[114]
Flavostatin	*Trimeserusus flavoviridis*	RGD	May-96	[49]
Bitistatin	*Bitis arietans*	RGD	October-97	[115]
Salmosin	*Agkistrodon Halys Brevicaudus*	RGD	July-98	[116]
Accutin	*Agkistrodon acutus*	RGD	November-98	[64]
EC3	*Echis carinatus*	VGD/MLD	April-99	[46]
Rhodocetin	*Calloselasma rhodostoma*	?	May-99	[117]
Jarastatin	*Bothrops jararaca*	RGD	September-99	[82]
EMF-10	*Eristicophis macmahoni*	RGD/MGD	September-99	[47]
EC6	*Echis carinatus*	MLD/RGD	October-00	[118]
Alternagin-C	*Bothrops alternatus*	ECD	December-00	[119]
Lebein	*Macrovipera lebetina*	RGD	May-01	[120]
Trimestatin	*Trimeresurus flavoviridis*	RGD	September-01	[121]
Piscivostatin	*Agkistrodon piscivorus piscivorus*	RGD/KGD	September-01	[121]
Saxatillin	*Gloydius saxatilis*	RGD	January-02	[66]
CC5	*Cerastes cereastes*	RGD	January-02	[88]
CC8	*Cerastes cereastes*	RGD/WRG	January-02	[88]
Ocellatusin	*Echis ocellatus*	RGD	February-02	[122]
Bothrasperin	*Bothrops asper*	RGD	March-03	[123]
Obtustatin	*Macrovipera lebetina*	KTS	May-03	[77]
EO4	*Echis ocellatus*		June-03	[124]
EO5	*Echis ocellatus*	MLD/VGD	June-03	[124]
VA6	*Vipera ammodytes*	RGD	June-03	[124]
VB7	*Vipera berus*	RGD/KGD	June-03	[124]
VLO4	*Vipera lebetina obtusa*		June-03	[124]
VLO5	*Vipera lebetina obtusa*	VGD/MLD	June-03	[124]
Adinbitor	*Agkistrodon halys brevicaudus stejneger*	RGD	June-04	[125]
Viperistatin	*Vipera palestinae*	KTS	November-04	[126]
Bothrostatin	*Bothrops jararaca*	RGD	April-05	[127]
Jerdostatin	*Trimeresurus jerdonii*	RTS	December-05	[128]
Lebestatin	*Macrovipera lebetina*	KTS	December-05	[59]
Mojastin-1 and -2	*Crotalus scutulatus scutulatus*	RGD	April-06	[129]
DisBa-01	*Bothrops alternatus*	RGD	October-07	[128]
Viplebedin-2	*Vipera lebetina*	VGD/MLD	July-09	[113]
Disintegrin protein	*Naja naja*	?	August-12	[130]
Disintegrin	*Atropoides mexicanus*	RGD	December-14	[61]
Sasaimin	*Cerrophidion sasai*	RGD	December-14	[61]
Simusmin	*Crotalus simus*	RGD	December-14	[61]
Tzabcanin	*Crotalus simus tzabcan*	RGD	September-15	[79]
Disintegrin_CC	*Cerastes cereastes*	RGD	December-17	[131]
Disintegrin	*Crotalus durissus collilineatus*	Non-RGD	October-18	[132]
Cerastategrin	*Cerastes cereastes*	RGD	September-20	[133]

Initially, disintegrins were studied for their inhibition of platelet aggregation due to the ability to interact with the transmembrane GPIIb/IIIa receptors (or αIIbβ3 integrin) present on the surface of platelets [39, 48-50]. Fibrinogen is a bivalent molecule capable of simultaneously binding to the activated GPIIb/IIIa receptor on two different platelets, forming bridges between the activated platelets [51-54]. Thus, disintegrins inhibit platelet aggregation by preventing the interaction of the αIIbβ3 integrin with fibrinogen.

Subsequently, in addition to their action on platelet receptors, many disintegrins have been isolated and characterized for their effects on other cells, demonstrating various biological functions, including interference with human neutrophil chemotaxis to sites of inflammation and tissue injury [55], antiparasitic activity [56], antiviral activity [57] and antitumor action through induction of apoptosis [50] and cytotoxicity [58], as well as inhibition of important steps in tumor development and progression, like adhesion [46, 59-63], angiogenesis [59, 64-67], migration [59, 62, 63, 68, 69] and metastasis [69-72]. 

### Structural characterization of snake venom disintegrins

**Figure 2.**
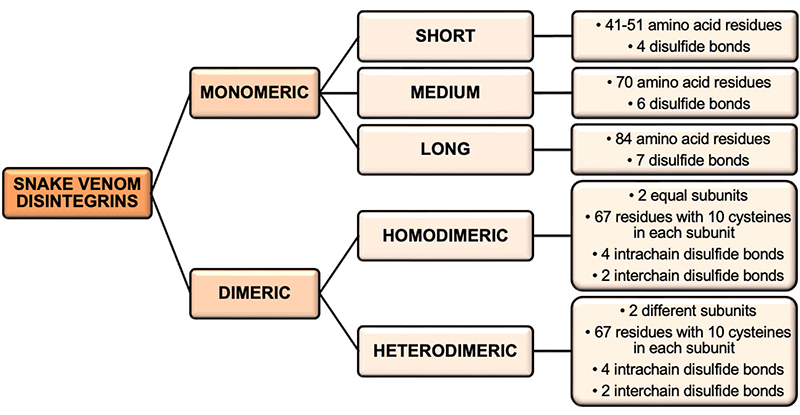
Structural classification of disintegrins.

Snake venom disintegrins can be structurally classified into two major groups: monomeric and dimeric (Figure 2). Monomeric disintegrins are composed of three classes [73]. The first class consists of short disintegrins with 41 to 51 amino acid residues and four disulfide bonds. The second class comprises medium disintegrins with approximately 70 amino acids and six disulfide bonds. The third class of monomeric disintegrins contains long disintegrins with about 84 residues and seven disulfide bridges [74]. The second group of disintegrins is the dimeric disintegrins, which are further classified as homo- or heterodimers when the subunits are identical or different, respectively [73]. The subunits of dimeric disintegrins are composed of around 67 residues with ten cysteines, which are involved in forming four intrachain and two interchain disulfide bonds [74].

**Figure 3.**
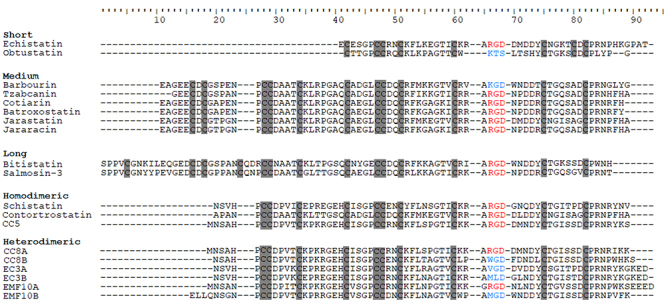
Multiple alignments among selected disintegrins from different structural classes. Cysteine residues are highlighted in gray. The integrin-binding RGD motif is represented in red, and non-RGD motifs are in blue.

These proteins are highly homologous, and this structural similarity is primarily associated with the alignment of cysteine residues [75]. Figure 3 shows the analysis of multiple sequence alignments of disintegrin domains from five different structural classes, including Echistatin [76], Obtustatin [77], Barbourin [78], Tzabcanin [79], Cotiarin [80], Batroxostatin [81], Jarastatin [82, 83], Jararacin [82-84], Bitistatin [85], Salmosin-3 [86], Schistatin [87], Contortrostatin [48], CC5 [88], CC8 [88], EC3 [46] and EMF10 [47], highlighting conserved cysteine residues (Figure 3).

**Figure 4.**
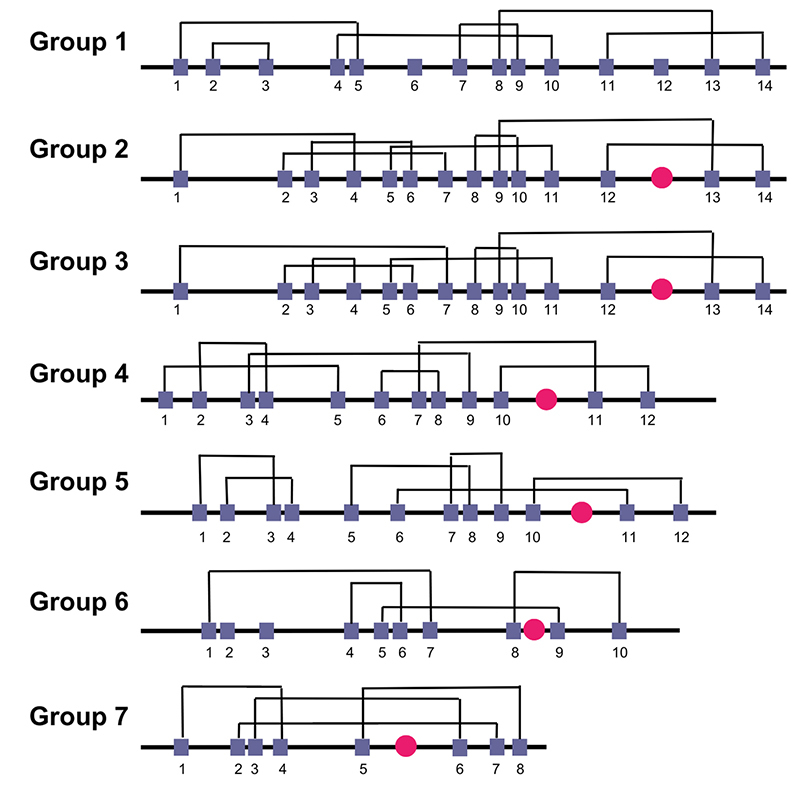
Disulfide bonding pattern for each group within the disintegrin family. (**Group 1:**) DAM/SVMP subfamily-like disintegrin domain proteins; (**Group 2:**) Bitistatin A-like disintegrins; (**Group 3:**) Bitistatin B-like disintegrins; (**Group 4:**) Kistrin-like disintegrins; (**Group 5:**) Salmosin-like disintegrins; (**Group 6:**) Dimeric disintegrins; (**Group 7:**) Short disintegrins. Purple squares indicate cysteine residues, while pink circle indicates the integrin-binding motif.

Regarding binding specificity, the correct pairing of cysteine residues is essential for exposing the motif that mediates the interaction with integrins and determining their inhibition [74]. In this context, the family of snake venom disintegrins can be divided into seven groups, each with a specific pattern of sequence and disulfide bond formation between cysteine residues (Figure 4). Group 1 includes the disintegrin-like domain of proteins from the ADAM/SVMP subfamily. Its disulfide pattern is defined as Cys1-Cys5, Cys2-Cys3, Cys4-Cys10, Cys7-Cys9, Cys8-Cys13, Cys11-Cys14, while Cys6 and Cys12 form connections with other domains of the protein. Group 2 consists of disintegrins similar to Bitistatin A, and Cys1-Cys4, Cys2-Cys7, Cys3-Cys6, Cys5-Cys11, Cys8-Cys10, Cys9-Cys13, Cys12-Cys14 characterize their disulfide pattern. Group 3 is formed by disintegrins similar to Bitistatin B, and their disulfide bond pattern consists of Cys1-Cys7, Cys2-Cys6, Cys3-Cys4, Cys5-Cys11, Cys8-Cys10, Cys9-Cys13, Cys12-Cys14. Group 4 consists of monomeric disintegrins similar to Kistrin, and the disulfide pattern of these molecules is Cys1-Cys5, Cys2-Cys4, Cys3-Cys9, Cys6-Cys8, Cys7-Cys11, Cys10-Cys12. Group 5 is the Salmosin group, also composed of monomeric disintegrins, and their disulfide pattern is Cys1-Cys3, Cys2-Cys4, Cys5-Cys8, Cys7-Cys9, Cys6-Cys11, Cys10-Cys12. Group 6 includes dimeric disintegrins, with an intrachain disulfide pattern characterized by Cys1-Cys7, Cys4-Cys6, Cys5-Cys9, Cys8-Cys10, while Cys2 and Cys3 form a disulfide bridge with the other subunit of the dimer. Lastly, group 7 comprises short disintegrins, and the disulfide pattern of these molecules can be described as Cys1-Cys4, Cys2-Cys7, Cys3-Cys6, and Cys5-Cys8 [89].

### Function and potential applications of snake venom disintegrins

**Figure 5.**
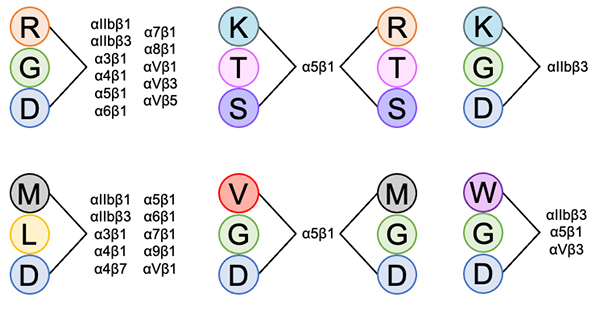
Interaction of snake venom disintegrins motifs with different integrins.

Snake venom disintegrins can selectively bind to integrins, which are strongly tied to the specific motifs found in their structure [90] (Figure 5). This way, during envenomation, they exhibit a wide array of functions, serving various crucial roles, like binds to platelet receptors, impeding their aggregation, and resulting in the onset of bleeding disorders [91]. Consequently, disintegrins contribute to disrupting hemostatic processes (Table 2).

**Table 2.** Snake venom disintegrins that can act on the hemostatic system.

Disintegrin (snake venom)	Motif	Integrins	Action	Ref.
Accutin (*Agkistrodon acutus*)	RGD	αIIbβ3	Inhibit human platelet aggregation induced by ADP, collagen, fibrinogen, thrombin and the thromboxane analogue U46619 Inhibit platelet aggregation of platelet-rich plasma	[134]
Albolabrin (*Trimeserusus albolabris*)	RGD	αIIbβ3	Block platelet-fibrinogen interaction Inhibit ADP-induced platelet aggregation of platelet-rich plasma	[111,135]
Applagin (*Agkistrodon piscivorus piscivorus*)	RGD	αIIbβ3	Block platelet aggregation induced by ADP, collagen, thrombin, and arachidonic acid	[110]
Barbourin (*Sistrurus miliarius barbouri*)	KGD	αIIbβ3	Inhibit fibrinogen to bind αIIbβ3 integrin	[78]
Basilicin (*Crotalus basilicus*)	RGD	αvβ3 α5β1 αIIbβ3	Inhibit platelet aggregation, adhesion to vitronectin, and fibrinogen to binding integrins	[136]
Bitistatin (*Bitis arietans*)	RGD	αIIbβ3	Block platelet-fibrinogen interaction Inhibit ADP-induced platelet aggregation of platelet-rich plasma	[135]
CC5 (*Cerastes cereastes*)	RGD	αIIbβ3	Inhibit ADP-induced platelet aggregation of platelet-rich plasma	[88]
CC8 (*Cerastes cereastes*)	RGD/WRG	αIIbβ3	Inhibit ADP-induced platelet aggregation of platelet-rich plasma	[88]
Cerastin (*Cerastes cereastes*)	RGD	αvβ3 α5β1 αIIbβ3	Inhibit platelet aggregation, adhesion to vitronectin, and fibrinogen to binding integrins	[136]
Cereberin (*Crotalus viridis cereberus*)	RGD	αvβ3 α5β1 αIIbβ3	Inhibit platelet aggregation, adhesion to vitronectin, and fibrinogen to binding integrins	[136]
Contortrostatin (*Agkistrodon contortrix contortrix*)	RGD	αIIbβ3	Inhibit ADP-induced platelet aggregation of platelet-rich plasma from humans, dogs and rabbits	[114]
Crotatoxin (*Crotalus atrox*)	RGD	αvβ3 α5β1 αIIbβ3	Inhibit platelet aggregation, adhesion to vitronectin, and fibrinogen to binding integrins	[136]
Cotiarin (*Bothrops cotiara*)	RGD	αvβ3 α5β1 αIIbβ3	Inhibit platelet aggregation, adhesion to vitronectin, and fibrinogen to binding integrins	[136]
Durissin (*Crotalus durissus durissus*)	RGD	αvβ3 α5β1 αIIbβ3	Inhibit platelet aggregation, adhesion to vitronectin, and fibrinogen to binding integrins	[136]
EC3 (*Echis carinatus*)	VGD/MLD	αIIbβ3	Inhibit fibrinogen to bind αIIbβ3 integrin	[137]
Echistatin (*Echis carinatus*)	RGD	αIIbβ3	Block platelet-fibrinogen interaction Inhibit ADP-induced platelet aggregation of platelet-rich plasma	[135]
Elegantin (*Trimeserusus elegans*)	RGD	αIIbβ3	Inhibit ADP-induced platelet aggregation of platelet-rich plasma	[111]
EMF-10 (*Eristicophis macmahoni*)	RGD/MGD	αIIbβ3	Inhibit ADP-induced platelet aggregation	[47]
Eristostatin (*Eristicophis macmahoni*)	RGD	αIIbβ3	Able to bind in ADP-, thrombin-induced, and resting platelet	[138]
Flavoridin (*Trimeserusus flavoviridis*)	RGD	αIIbβ3	Block platelet-fibrinogen interaction Inhibit ADP-induced platelet aggregation of platelet-rich plasma	[135]
Jararacin (*Bothrops jararaca*)	RGD	αvβ3 α5β1 αIIbβ3	Inhibit ADP- and thrombin-induced platelet aggregation Inhibit adhesion to vitronectin, and fibrinogen to binding integrins	[136,139]
Jarastatin (*Bothrops jararaca*)	RGD	αIIbβ3	Inhibit ADP- and thrombin-induced platelet aggregation	[139]
Jerdostatin (*Trimeresurus jerdonii*)	RTS	αIIbβ3	Inhibit fibrinogen to bind αIIbβ3 integrin	[140]
Lachesin (*Lachesis mutus*)	RGD	αvβ3 α5β1 αIIbβ3	Inhibit platelet aggregation, adhesion to vitronectin, and fibrinogen to binding integrins	[136]
Lebein (*Macrovipera lebetina*)	RGD	?	Inhibit ADP-induced platelet aggregation of platelet-rich plasma	[120]
Lutosin (*Crotalus viridis lutosus*)	RGD	αvβ3 α5β1 αIIbβ3	Inhibit platelet aggregation, adhesion to vitronectin, and fibrinogen to binding integrins	[136]
Mojastin-1 and -2 (*Crotalus scutulatus scutulatus*)	RGD	α5β1	Inhibit ADP-induced platelet aggregation of whole blood	[129]
Molossin (*Crotalus molossus molossus*)	RGD	αvβ3 α5β1 αIIbβ3	Inhibit platelet aggregation, adhesion to vitronectin, and fibrinogen to binding integrins	[136]
Multisquamatin (*Echis multisquamatus*)	RGD	αIIbβ3	Inhibit ADP-induced platelet aggregation of platelet-rich plasma from humans, dogs and rabbits	[114]
Rhodocetin (*Calloselasma rhodostoma*)	?	?	Inhibit collagen-induced platelet aggregation	[117]
Saxatillin (*Gloydius saxatilis*)	RGD	αIIbβ3	Inhibit the interaction of integrins and fibrinogen Inhibit ADP-induced platelet aggregation	[66]
Triflavin (*Protobothrops flavoviridis*)	RGD	αIIbβ3	Inhibit ADP-induced and resting platelet	[113]
Trigramin (*Trimeresurus gramineus*)	RGD	αIIbβ3	Inhibit the interaction of ADP-induced platelet and fibrinogen Inhibit chymotrypsin-treated platelet aggregation Bind to resting platelet	[44]
Viplebedin-2 (*Vipera lebetina*)	VGD/MLD	?	Inhibit ADP- and collagen-induced platelet aggregation Inhibit platelet adhesion	[137]
Viridin (*Crotalus viridis viridis*)	RGD	αvβ3 α5β1 αIIbβ3	Inhibit platelet aggregation, adhesion to vitronectin, and fibrinogen to binding integrins	[136]

Some snake venom disintegrins can inhibit bone resorption *in vitro* [92] and can also be used as a diagnostic tool. An example, we cite bitistatin, which can potentially be used in molecular imaging of thromboembolic diseases [93].

It has also been demonstrated that disintegrins can interfere with the chemotaxis of human neutrophils to sites of inflammation and tissue injury [55] and exhibit antiparasitic activity against *Leishmania infantum* promastigotes [56].

Intriguingly, certain disintegrins have demonstrated notable anti-tumor and anti-angiogenic properties (Table 3). This remarkable feature opens up new possibilities for their utilization as potential therapeutic agents in cancer treatment, and by targeting tumor growth and impeding blood vessel formation, these disintegrins exhibit promising potential in medical research and innovation.

**Table 3.** Discovery of snake venom disintegrins that can act as anticancer agents.

Disintegrin (snake venom)	Motif	Cell line (cancer type)	Integrins	Action	Ref.
Accutin (*Agkistrodon acutus*)	RGD	HUVEC (human non-cancer cell)	αvβ3	Induce apoptosis Inhibit angiogenesis *in vitro* and *in vivo*	[141]
Albolabrin (*Trimeserusus albolabris*)	RGD	B16-F10 (murine melanoma)	α5β1 αvβ3 α6β1	Inhibit cell-matrix attachment in vitro Inhibit metastasis of tumor cells	[142]
Alternagin-C (*Bothrops alternatus*)	ECD	HUVEC (human non-cancer cell) MDA-MB-231 (human breast cancer) HMEC-1 (human cells from tumor microenvironment) Human fibroblasts	α2β1	Modulates cell adhesion, migration and proliferation Inhibit adhesion, viability and migration of VEGF-induced cell Inhibit angiogenesis *in vitro* Infer in tumor progression	[143-145]
Barbourin (*Sistrurus miliarius barbouri*)	KGD	B16-F10 (murine melanoma)	αvβ3 αvβ1	Inhibit cell adhesion	[146]
Bitistatin (*Bitis arietans*)	RGD	HUVEC (human non-cancer cell)	αvβ3	Inhibit cell adhesion	[147]
CC5 (*Cerastes cereastes*)	RGD	A5 (murine non-cancer cell) JY (human lymphoblastoid cell) Κ562 (human myelogenous leukemia) CHO K1 (murine non-cancer cell)	α5β1 αvβ3	Inhibit cell adhesion	[88]
CC8 (*Cerastes cereastes*)	RGD/WRG	A5 (murine non-cancer cell) JY (human lymphoblastoid cell) Κ562 (human myelogenous leukemia) CHO K1 (murine non-cancer cell)	α5β1 αvβ3	Inhibit cell adhesion	[88]
Contortrostatin (*Agkistrodon contortrix contortrix*)	RGD	M24 met (human metastatic melanoma)	α5β1 αvβ1	Inhibit cell adhesion *in vitro* Inhibit lung colonization *in vivo*	[148]
DisBa-01 (*Bothrops alternatus*)	RGD	HMEC-1 (human non-cancer cell) MDA-MB-231 (human breast cancer) B16-F10 (murine melanoma)	αvβ3	Inhibit angiogenesis Inhibit cell adhesion and proliferation	[149]
Disintegrin (*Crotalus durissus collilineatus*)	Non-RGD	MDA-MB-231 (human breast cancer)	?	Inhibit cell migration	[132]
EC3 (*Echis carinatus*)	VGD/MLD	A5 (murine non-cancer cell) VNRC3 (murine non-cancer cell) CHO (murine non-cancer cell) JY (human lymphoblastoid cell) Κ562 (human myelogenous leukemia) Jurkat (human acute T cell leukemia) CHO K1 (murine non-cancer cell) RPMI886 (human chronic myelogenous leukaemia)	αIIbβ3 α5β1 αvβ3 α4β1 α4β7	Inhibit cell adhesion	[46]
EC6 (*Echis carinatus*)	MLD/RGD	A5 (murine non-cancer cell) Κ562 (human myelogenous leukemia) Jurkat (human acute T cell leukemia)	α5β1 α4β1	Inhibit cell adhesion	[118]
Echistatin (*Echis carinatus*)	RGD	A5 (murine non-cancer cell) JY (human lymphoblastoid cell) Κ562 (human myelogenous leukemia) SW480 (human colon adenocarcinoma) Jurkat (human acute T cell leukemia)	α5β1 αvβ3	Inhibit cell adhesion Inhibit angiogenesis	[150]
EMF-10 (*Eristicophis macmahoni*)	RGD/MGD	Κ562 (human myelogenous leukemia)	α5β1	Inhibit cell adhesion	[47]
EO5 (*Echis ocellatus*)	MLD/VGD	A5 (murine non-cancer cell) Κ562 (human myelogenous leukemia) Jurkat (human acute T cell leukemia)	α4β1	Blocked cell adhesion	[124]
Eristostatin (*Eristicophis macmahoni*)	RGD	A375 (human malignant melanoma) HT1080 (human fibrosarcoma)	αIIbβ3 α5β1 αvβ3	Inhibit cell adhesion	[151]
Jerdostatin (*Trimeresurus jerdonii*)	RTS	JY (human lymphoblastoid cell) Κ562 (human myelogenous leukemia) SW480 (human colon adenocarcinoma) Jurkat (human acute T cell leukemia)	αIIbβ3 α5β1 α1β1 α2β1 α6β1 αvβ3 α4β1 α9β1	Inhibit cell adhesion	[140]
Lebein (*Macrovipera lebetina*)	RGD	LS174, HCT116, and HT29 (human colon adenocarcinoma) SK-MEL-28 and LU-1205 (human melanoma)	α5β1 αvβ3	Induce apoptosis Inhibit cell migration and adhesion Inhibit angiogenesis by down-regulating VEGF and NRP1 Expression	[152,153]
Lebestatin (*Macrovipera lebetina*)	KTS	CHO (murine non-cancer cell) HT29-D4 (human colonic adenocarcinoma) HT1080 (human fibrosarcoma) Κ562 (human myelogenous leukemia) IGROV1 (human ovarian adenocarcinoma) HMEC-1 (human non-cancer cell) PC12 (rat pheochromocytoma)	α1β1	Inhibit cell migration and adhesion Inhibit angiogenesis	[59]
Mojastin-1 and -2 (*Crotalus scutulatus scutulatus*)	RGD	BXPC-3 (human pancreatic adenocarcinoma)	α3β1	Inhibit cell proliferation, migration and adhesion Induce apoptosis	[154]
Obtustatin (*Macrovipera lebetina*)	KTS	A5 (murine non-cancer cell) Κ562 (human myelogenous leukemia) Jurkat (human acute T cell leukemia)	α1β1	Inhibit angiogenesis *in vivo*	[77,150]
Purpureomaculin (*Trimeresurus purpureomaculatus*)	RGD	MCF-7 (human breast adenocarcinoma)	αvβ5	Inhibit cell growth	[155]
Rhodocetin (*Calloselasma rhodostoma*)	?	HT1080 (human fibrosarcoma)	α2β1	Inhibit cell adhesion and migration	[156]
Rhodostomin (*Calloselasma rhodostoma*)	RGD	B16-F10 (murine melanoma) HUVEC (human non-cancer cell)	αvβ3	Inhibit angiogenesis Suppress tumor growth *in vivo* Inhibit cell proliferation	[157]
Saxatillin (*Gloydius saxatilis*)	RGD	HUVEC and SMC (human non-cancer cells) MDAH2774 (human ovarian cancer cells)	αvβ3	Inhibit cell proliferation, migration and adhesion Inhibit angiogenesis Inhibit tumor metastasis	[66,158,159]
Triflavin (*Protobothrops flavoviridis*)	RGD	B16-F10 (murine melanoma)	αIIbβ3	Inhibit cell adhesion	[160]
Tzabcanin (*Crotalus simus tzabcan*)	RGD	A-357 (human malignant melanoma) Colo-205 (human colorectal adenocarcinoma) MCF-7 (human breast adenocarcinoma) A-549 (human lung adenocarcinoma)	αvβ3	Inhibit cell migration and adhesion	[79,161]
VA6 (*Vipera ammodytes*)	RGD	A5 (murine non-cancer cell) Κ562 (human myelogenous leukemia) Jurkat (human acute T cell leukemia)	α5β1	Inhibit cell adhesion	[124]
VB7 (*Vipera berus*)	RGD/KGD	A5 (murine non-cancer cell) Κ562 (human myelogenous leukemia) Jurkat (human acute T cell leukemia)	α5β1	Inhibit cell adhesion	[124]
Viperistatin (*Vipera palestinae*)	KTS	A5 (murine non-cancer cell) JY (human lymphoblastoid cell) Κ562 (human myelogenous leukemia) SW480 (human colon adenocarcinoma)	α1β1	Inhibit cell adhesion	[126]
VLO5 (*Vipera lebetina obtusa*)	VGD/MLD	A5 (murine non-cancer cell) Κ562 (human myelogenous leukemia) Jurkat (human acute T cell leukemia)	α4β1	Block cell adhesion	[124]

### Snake venom disintegrins: from lab bench to market

Animal venoms are rich mixtures of components that may have important pharmacological actions. Many of these components have already been extensively studied to become drugs, and after approval by the Food and Drug Administration (FDA), turned into widely used molecules [94].

A very important example of a drug derived from animal toxins is captopril (Capoten®, Bristol-Myers Squibb, New York, NY, EUA), which is widely used against hypertension [95]. This was the first animal-derived drug approved by the FDA in 1981, which mechanism is responsible for inhibiting the angiotensin-converting enzyme (ACE). Thus, the production of angiotensin II is also inhibited, reducing hypertension effects, and increasing the hypotensive action of bradykinin, known as a bradykinin potentiating factor (BPF) [96-99]. Although it is a very effective natural molecule, the captopril used in medicaments is a synthetic molecule based on the miniaturization of the original molecule and chemically modified to be administered orally [94, 100]. In sequence, in 1985, the FDA approved Enalapril (Vasotec®, Merck, Darmstadt, Germany), which was also used to treat hypertension and congestive heart failure [94, 101].

Some disintegrins have been extensively studied and are nowadays FDA-approved drugs as well. Tirofiban (Aggrastat®, Medicure International, Inc., Winnipeg, Manitoba, Canada) is also a synthetic drug based on the RGD domain of echistatin from *Echis carinatus* [102]. Furthermore, it has a chemical modification that increases its interaction with platelet glycoproteins, specifically with their GPIIb/IIIa receptors [76]. Thus, this drug can inhibit platelet aggregation and other thrombotic actions due to its competition with fibrinogen for the recognition site of the RGD domain in the GPIIb/IIIa receptor [102, 103]. Tirofiban was approved by the FDA in 1998 as a treatment for acute coronary syndrome [104].

Another antiplatelet compound, Eptifibatide (Integrilin®, Millennium Pharmaceuticals, Inc.), was also approved by the FDA in 1998, and licensed in 2005, to Schering-Plough [94]. Its development coincided with the research for the synthetic peptide analogs of barbourin, a disintegrin from *Sistrurus miliarius barbouri* [78]. The conservative substitution of arginine (R) amino acids with lysine (K) in barbourin enhances its specificity towards the platelet glycoprotein complex GPIIb/IIIa compared to other disintegrins containing the RGD motif [78]. However, this specificity may also be influenced by the size of the peptide ring formed by disulfide bridges and the amino acids near the KGD domain. As a result, new peptides have been synthesized for potential clinical use, such as Eptifibatide, a synthetic heptapeptide that is more resistant to proteolysis [105-107].

**Figure 6.**
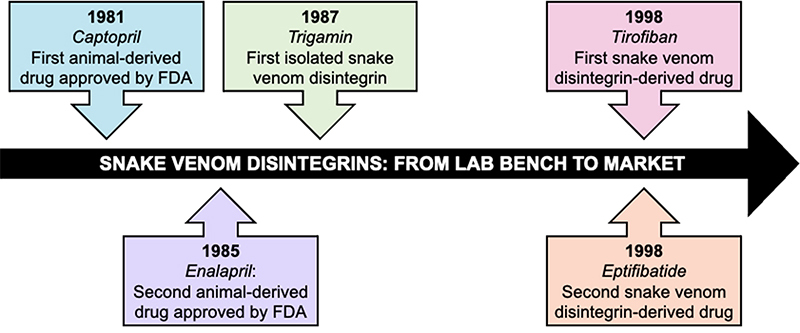
Timeline of snake venom disintegrins, from the beginning of disintegrins’ saga in Toxinology until their FDA approval.

Since the approval of the first venom-derived drug and the beginning of disintegrins’ saga in Toxinology [44], it took over 10 years of research and effort for the first medication derived from snake venom disintegrins also to be approved (Figure 6). However, it was already known that venoms and their components could cause modifications in the human body, and their applicability in clinical settings had been recognized.

Currently, a product based on snake venom toxins has been attracting attention: Heterologous Fibrin Sealant. This sealant is composed of a thrombin-like enzyme from *Crotalus durissus terrificus* venom and fibrinogen-rich cryoprecipitate extracted from the blood of *Bubalus bubalis buffaloes*. It can be used for the treatment of chronic venous ulcers, as demonstrated in phase I/II clinical trials, highlighting its effectiveness and safety [108]. While there are currently no clinical studies using snake venom disintegrins, human disintegrins, especially ADAMs, have been targeted for the therapy of other pathological conditions in clinical trials, such as cirrhosis and portal hypertension (NCT04267406), epithelial dysfunction (NCT00898859), idiopathic pulmonary arterial hypertension and chronic thromboembolic pulmonary hypertension (NCT05478226), among others [109].

## Conclusion

Snake venom disintegrins’ saga was started in 1987 and classified these molecules as small peptides that can inhibit the function of integrins, which are cell surface receptors involved in various cellular processes like cell adhesion, migration, and signaling. Integrins are important for cell adhesion to extracellular matrix proteins, mediating cell-cell interactions, and interfering in integrin-mediated processes, as snake venom disintegrins can have various effects on cells and tissues.

Among their unique properties, snake venom disintegrins can inhibit platelet aggregation, *i.e.*, bind to integrins on platelets, preventing their aggregation and potentially disrupting the clotting process. Consequently, two important antiplatelet drugs were based on disintegrins from snake venoms, and they are on the market nowadays.

Moreover, snake venom disintegrins have shown anti-cancer properties by targeting integrins that are overexpressed in specific cancer cells and blocking integrin-mediated signaling pathways. These disintegrins can also inhibit tumor growth and metastasis. Notably, although snake venom disintegrins possess therapeutic potential, they exhibit high potency and can manifest toxicity. Thus, rigorous investigation is required before contemplating snake venom disintegrin use in medical applications.

## References

[B1] Munawar A, Ali S, Akrem A, Betzel C (2018). Snake Venom Peptides: Tools of Biodiscovery. Toxins.

[B2] Casewell NR, Jackson TNW, Laustsen AH, Sunagar K (2020). Causes and Consequences of Snake Venom Variation. Trends Pharmacol Sci.

[B3] Westeen EP, Durso AM, Grundler MC, Rabosky DL, Davis Rabosky AR (2020). What makes a fang? Phylogenetic and ecological controls on tooth evolution in rear-fanged snakes. BMC Evol Biol.

[B4] Chippaux JP (2017). Snakebite envenomation turns again into a neglected tropical disease!. J Venom Anim Toxins incl Trop Dis.

[B5] World Health Organization (2021). Snakebite envenoming.

[B6] Gutiérrez JM, Calvete JJ, Habib AG, Harrison RA, Williams DJ, Warrell DA (2017). Snakebite envenoming. Nat Rev Dis Primers.

[B7] El-Aziz Mohamed Abd, Garcia Soares A, Stockand JD (2019). Snake Venoms in Drug Discovery: Valuable Therapeutic Tools for Life Saving. Toxins (Basel).

[B8] Calvete JJ, Juárez P, Sanz L (2007). Snake venomics. Strategy and applications. J Mass Spectrom.

[B9] Lomonte B, Fernández J, Sanz L, Angulo Y, Sasa M, Gutiérrez JM, Calvete JJ (2014). Venomous snakes of Costa Rica: Biological and medical implications of their venom proteomic profiles analyzed through the strategy of snake venomics. J Proteomics.

[B10] Tasoulis T, Pukala TL, Isbister GK (2022). Investigating Toxin Diversity and Abundance in Snake Venom Proteomes. Front Pharmacol.

[B11] Tasoulis T, Isbister G (2017). A Review and Database of Snake Venom Proteomes. Toxins (Basel).

[B12] Arruda Macedo J, Fox J, Souza Castro M (2015). Disintegrins from Snake Venoms and their Applications in Cancer Research and Therapy. Curr Protein Pept Sci.

[B13] Lucena S, Castro R, Lundin C, Hofstetter A, Alaniz A, Suntravat M, Sánchez EE (2015). Inhibition of pancreatic tumoral cells by snake venom disintegrins. Toxicon.

[B14] Cesar PHS, Braga MA, Trento MVC, Menaldo DL, Marcussi S (2019). Snake Venom Disintegrins: An Overview of their Interaction with Integrins. Curr Drug Targets.

[B15] Bianconi D, Unseld M, Prager G (2016). Integrins in the Spotlight of Cancer. Int J Mol Sci.

[B16] Takada Y, Ye X, Simon S (2007). The integrins. Genome Biol.

[B17] Mezu-Ndubuisi OJ, Maheshwari A (2021). The role of integrins in inflammation and angiogenesis. Pediatr Res.

[B18] Arnaout MA, Mahalingam B, Xiong JP (2005). Integrin structure allostery, and bidirectional signaling. Annu Rev Cell Dev BioLife-Saving.

[B19] Morse EM, Brahme NN, Calderwood DA (2014). Integrin Cytoplasmic Tail Interactions. Biochemistry.

[B20] Tsuji T (2004). Physiological and Pathological Roles of α3β1 Integrin. J Membr Biol.

[B21] Bachmann M, Kukkurainen S, Hytönen VP, Wehrle-Haller B (2019). Cell Adhesion by Integrins. Physiol Rev.

[B22] Koivisto L, Heino J, Häkkinen L, Larjava H (2014). Integrins in Wound Healing. Adv Wound Care.

[B23] Lilja J, Ivaska J (2018). Integrin activity in neuronal connectivity. J Cell Sci.

[B24] Park YK, Goda Y (2016). Integrins in synapse regulation. Nat Rev Neurosci.

[B25] Conroy KP, Kitto LJ, Henderson NC (2016). αv integrins: key regulators of tissue fibrosis. Cell Tissue Res.

[B26] Finney AC, Stokes KY, Pattillo CB, Orr AW (2017). Integrin signaling in atherosclerosis. Cell Mol Life Sci.

[B27] Seguin L, Desgrosellier JS, Weis SM, Cheresh DA (2015). Integrins and cancer: regulators of cancer stemness, metastasis, and drug resistance. Trends Cell Biol.

[B28] Desgrosellier JS, Cheresh DA (2010). Integrins in cancer: biological implications and therapeutic opportunities. Nat Rev Cancer.

[B29] Niu J, Li Z (2017). The roles of integrin αvβ6 in cancer. Cancer Lett.

[B30] Hamidi H, Ivaska J (2018). Every step of the way: integrins in cancer progression and metastasis. Nat Rev Cancer.

[B31] Adorno-Cruz V, Liu H (2019). Regulation and functions of integrin α2 in cell adhesion and disease. Genes Dis.

[B32] Lazarovici P, Marcinkiewicz C, Lelkes PI (2019). From Snake Venom’s Disintegrins and C-Type Lectins to Anti-Platelet Drugs. Toxins (Basel).

[B33] Markland FS, Swenson S (2013). Snake venom metalloproteinases. Toxicon.

[B34] Takeda S (2016). ADAM and ADAMTS Family Proteins and Snake Venom Metalloproteinases: A Structural Overview. Toxins (Basel).

[B35] Sanz L, Harrison RA, Calvete JJ (2012). First draft of the genomic organization of a PIII-SVMP gene. Toxicon.

[B36] Zychar BC, Clissa PB, Carvalho E, Alves AS, Baldo C, Faquim-Mauro EL, Gonçalves LRC (2021). Modulation of Adhesion Molecules Expression by Different Metalloproteases Isolated from Bothrops Snakes. Toxins (Basel).

[B37] Casewell NR (2012). On the ancestral recruitment of metalloproteinases into the venom of snakes. Toxicon.

[B38] Stone AL, Kroeger M, Sang QXA (1999). Structure-Function Analysis of the ADAM Family of Disintegrin-Like and Metalloproteinase-Containing Proteins (Review). J Protein Chem.

[B39] Okuda D, Koike H, Morita T (2002). A New Gene Structure of the Disintegrin Family: A Subunit of Dimeric Disintegrin Has a Short Coding Region. Biochemistry.

[B40] Juarez P, Comas I, Gonzalez-Candelas F, Calvete JJ (2008). Evolution of Snake Venom Disintegrins by Positive Darwinian Selection. Mol Biol Evol.

[B41] Calvete JJ, Kini RM, Clemetson KJ, Markland FS, McLane MA, Morita T (2010). Toxins and Hemostasis.

[B42] Moura-da-Silva A, Almeida M, Portes-Junior J, Nicolau C, Gomes-Neto F, Valente R (2016). Processing of Snake Venom Metalloproteinases: Generation of Toxin Diversity and Enzyme Inactivation. Toxins (Basel).

[B43] Kini RM, Evans HJ (1992). Structural domains in venom proteins: Evidence that metalloproteinases and nonenzymatic platelet aggregation inhibitors (disintegrins) from snake venoms are derived by proteolysis from a common precursor. Toxicon.

[B44] Huang TF, Holt JC, Lukasiewicz H, Niewiarowski S (1987). Trigramin. A low molecular weight peptide inhibiting fibrinogen interaction with platelet receptors expressed on glycoprotein IIb-IIIa complex. J Biol Chem.

[B45] Gould RJ, Polokoff MA, Friedman PA, Huang T-F, Holt JC, Cook JJ, Niewiarowski S (1990). Disintegrins: A Family of Integrin Inhibitory Proteins from Viper Venoms. Proc Soc Exp Biol Med.

[B46] Marcinkiewicz C, Calvete JJ, Marcinkiewicz MM, Raida M, Vijay-Kumar S, Huang Z, Lobb RR, Niewiarowski S (1999). EC3, a Novel Heterodimeric Disintegrin from Echis carinatus Venom, Inhibits α4 and α5 Integrins in an RGD-independent Manner. J Biol Chem.

[B47] Marcinkiewicz C, Calvete JJ, Vijay-Kumar S, Marcinkiewicz MM, Raida M, Schick P, Lobb RR, Niewiarowski S (1999). Structural and Functional Characterization of EMF10, a Heterodimeric Disintegrin from Eristocophis macmahoni Venom That Selectively Inhibits α5β1 Integrin. Biochemistry.

[B48] Trikha Mohit, De Clerck YA, Francis S (1994). Markland. Contortrostatin, a Snake Venom Disintegrin, Inhibits β1 Integrin-mediated Human Metastatic Melanoma Cell Adhesion and Blocks Experimental Metastasis. Cancer Res.

[B49] Kawasaki T, Sakai Y, Taniuchi Y, Sato K, Maruyama K, Shimizu M, Kaku S, Yano S, Inagaki O, Tomioka K, Yanagisawa I, Takenaka T (1996). Biochemical characterization of a new disintegrin, flavostatin, isolated from Trimeresurus flavoviridis venom. Biochimie.

[B50] Yeh CH, Peng HC, Yih JB, Huang TF (1998). A new short chain RGD-containing disintegrin, accutin, inhibits the common pathway of human platelet aggregation. Biochim Biophys Acta.

[B51] Nieswandt B, Varga-Szabo D, Elvers M (2009). Integrins in platelet activation. J Thromb Haemost.

[B52] Austin SK (2017). Haemostasis. Medicine.

[B53] McFadyen JD, Schaff M, Peter K (2018). Current and future antiplatelet therapies: emphasis on preserving haemostasis. Nat Rev Cardiol.

[B54] Bledzka K, Qin J, Plow EF (2019). Platelets.

[B55] Mariano-Oliveira A, Coelho ALJ, Terruggi CHB, Selistre-de-Araújo HS, Barja-Fidalgo C, De Freitas MS (2003). Alternagin-C, a nonRGD-disintegrin, induces neutrophil migration via integrin signaling: Effects of alternagin-C on neutrophil functions. Eur J Biochem.

[B56] Allane D, Oussedik-Oumehdi H, Harrat Z, Seve M, Laraba-Djebari F (2018). Isolation and characterization of an anti-leishmanial disintegrin from Cerastes cerastes venom. J Biochem Mol Toxicol.

[B57] Hubbard S, Choudhary S, Maus E, Shukla D, Swenson S, Markland FS, Tiwari V (2012). Contortrostatin, a Homodimeric Disintegrin Isolated from Snake Venom Inhibits Herpes Simplex Virus Entry and Cell Fusion. Antivir Ther.

[B58] Hailey S, Adams E, Penn R, Wong A, McLane MA (2013). Effect of the disintegrin eristostatin on melanoma-natural killer cell interactions. Toxicon.

[B59] Olfa K-Z, José L, Salma D, Amine B, Najet SA, Nicolas A, Maxime L, Raoudha Z, Kamel M, Jacques M, Jean-Marc S, Mohamed EA, Naziha Marrakchi (2005). Lebestatin, a disintegrin from Macrovipera venom, inhibits integrin-mediated cell adhesion, migration and angiogenesis. Lab Invest.

[B60] Sánchez EE, Rodríguez-Acosta A, Palomar R, Lucena SE, Bashir S, Soto JG, Pérez JC (2009). Colombistatin: a disintegrin isolated from the venom of the South American snake (Bothrops colombiensis) that effectively inhibits platelet aggregation and SK-Mel-28 cell adhesion. Arch Toxicol.

[B61] Ângulo Y, Castro A, Lomonte B, Rucavado A, Fernández J, Calvete JJ, Gutiérrez JM (2014). Isolation and characterization of four medium-size disintegrins from the venoms of Central American viperid snakes of the genera Atropoides, Bothrops, Cerrophidion and Crotalus. Biochimie.

[B62] Saviola AJ, Burns PD, Mukherjee AK, Mackessy SP (2016). The disintegrin tzabcanin inhibits adhesion and migration in melanoma and lung cancer cells. Int J Biol Macromol.

[B63] Montealegre-Sánchez L, Gimenes SNC, Lopes DS, Teixeira SC, Solano-Redondo L, De Melo Rodrigues V, Jiménez-Charris E (2019). Antitumoral Potential of Lansbermin-I, a Novel Disintegrin from Porthidium lansbergii lansbergii Venom on Breast Cancer Cells. Curr Top Med Chem.

[B64] Yeh CH, Peng H-C, Huang T-F (1998). Accutin, a New Disintegrin, Inhibits Angiogenesis In Vitro and In Vivo by Acting as Integrin αvβ3 Antagonist and Inducing Apoptosis. Blood.

[B65] Kang IC, Lee YD, Kim DS (1999). A Novel Disintegrin Salmosin Inhibits Tumor Angiogenesis. Cancer Res.

[B66] Hong SY, Koh YS, Chung KH, Kim DS (2002). Snake venom disintegrin, saxatilin, inhibits platelet aggregation, human umbilical vein endothelial cell proliferation, and smooth muscle cell migration. Thromb Res.

[B67] Zhou Q, Nakada MT, Arnold C, Shieh KY, Markland FS (1999). Contortrostatin, a dimeric disintegrin from Agkistrodon contortrix contortrix, inhibits angiogenesis. Angiogenesis.

[B68] Tian J, Paquette-Straub C, Sage EH, Funk SE, Patel V, Galileo D, McLane MA (2007). Inhibition of melanoma cell motility by the snake venom disintegrin eristostatin. Toxicon.

[B69] Galán JA, Sánchez EE, Rodríguez-Acosta A, Soto JG, Bashir S, McLane MA, Paquette-Straub C, Pérez JC (2008). Inhibition of lung tumor colonization and cell migration with the disintegrin crotatroxin 2 isolated from the venom of Crotalus atrox. Toxicon.

[B70] Danen EHJ, Marcinkiewicz C, Cornelissen IM, Van Kraats AA, Pachter JA, Ruiter DJ, Niewiarowski S, van Muijen GN (1998). The Disintegrin Eristostatin Interferes with Integrin α4β1 Function and with Experimental Metastasis of Human Melanoma Cells. Exp Cell Res.

[B71] Kang IC, Kim DS, Jang Y, Chung KH (2000). Suppressive Mechanism of Salmosin, a Novel Disintegrin in B16 Melanoma Cell Metastasis. Biochem Biophys Res Commun.

[B72] McLane MA, Kuchar MA, Brando C, Santoli D, Paquette-Straub CA, Miele ME (2001). New Insights on Disintegrin-Receptor Interactions: Eristostatin and Melanoma Cells. Haemostsis.

[B73] Walsh EM, Marcinkiewicz C (2011). Non-RGD-containing snake venom disintegrins, functional and structural relations. Toxicon.

[B74] Assumpcao TCF, Ribeiro JMC, Francischetti IMB (2012). Disintegrins from Hematophagous Sources. Toxins (Basel).

[B75] Calvete JJ (2013). The continuing saga of snake venom disintegrins. Toxicon.

[B76] Gan ZR, Gould RJ, Jacobs JW, Friedman PA, Polokoff MA (1988). Echistatin. A potent platelet aggregation inhibitor from the venom of the viper, Echis carinatus. J Biol Chem.

[B77] Marcinkiewicz C, Weinreb PH, Calvete JJ, Kisiel DG, Mousa SA, Tuszynski GP, Lobb RR (2003). Obtustatin: a potent selective inhibitor of alpha1beta1 integrin in vitro and angiogenesis in vivo. Cancer Res.

[B78] Scarborough RM, Rose JW, Hsu MA, Phillips DR, Fried VA, Campbell AM, l Nannizzi, Charo IF (1991). Barbourin. A GPIIb-IIIa-specific integrin antagonist from the venom of Sistrurus m. barbouri. J Biol Chem.

[B79] Saviola AJ, Modahl CM, Mackessy SP (2015). Disintegrins of Crotalus simus tzabcan venom: Isolation, characterization and evaluation of the cytotoxic and anti-adhesion activities of tzabcanin, a new RGD disintegrin. Biochimie.

[B80] Tashima AK, Sanz L, Camargo ACM, Serrano SMT, Calvete JJ (2008). Snake venomics of the Brazilian pitvipers Bothrops cotiara and Bothrops fonsecai. Identification of taxonomy markers. J Proteomics.

[B81] Rucinski B, Niewiarowski S, Holt JC, Soszka T, Knudsen KA (1990). Batroxostatin, an Arg-Gly-Asp-containing peptide from Bothrops atrox, is a potent inhibitor of platelet aggregation and cell interaction with fibronectin. Biochim Biophys Acta.

[B82] Coelho ALJ, de Freitas MS, Oliveira-Carvalho AL, Moura-Neto V, Zingali RB, Barja-Fidalgo C (1999). Effects of Jarastatin, a Novel Snake Venom Disintegrin, on Neutrophil Migration and Actin Cytoskeleton Dynamics. Expl Cell Res.

[B83] Wermelinger LS, Geraldo RB, Frattani FS, Rodrigues CR, Juliano MA, Castro HC, Zingali RB (2009). Integrin inhibitors from snake venom: Exploring the relationship between the structure and activity of RGD-peptides. Arch Biochem Biophys.

[B84] Scarborough RM, Rose JW, Naughton MA, Phillips DR, Nannizzi L, Arfsten A, Campbell AM, Charo IF (1993). Characterization of the integrin specificities of disintegrins isolated from American pit viper venoms. J Biol Chem.

[B85] Shebuski RJ, Ramjit DR, Bencen GH, Polokoff MA (1989). Characterization and Platelet Inhibitory Activity of Bitistatin, a Potent Arginine-Glycine-Aspartic Acid-Containing Peptide from the Venom of the Viper Bitis arietans. J Biol Chem.

[B86] Park Dongsu, Kang Incheol, Kim Hakdai, Chung Kwanghoe, Kim Doo-sik, Yun Yungdae (1998). Cloning and Characterization of Novel Disintegrins from Agkistrodon halys Venom. Mol Cells.

[B87] Bilgrami S, Tomar S, Yadav S, Kaur P, Kumar J, Jabeen T, Sharma S, Singh TP (2004). Crystal Structure of Schistatin, a Disintegrin Homodimer from Saw-scaled Viper (Echis carinatus) at 2.5Å Resolution. J Mol Biol.

[B88] Calvete JJ, Fox JW, Agelan A, Niewiarowski S, Marcinkiewicz C (2002). The Presence of the WGD Motif in CC8 Heterodimeric Disintegrin Increases Its Inhibitory Effect on αIIbβ3, αvβ3, and α5β1 Integrins. Biochemistry.

[B89] Vasconcelos AA, Estrada JC, David V, Wermelinger LS, Almeida FCL, Zingali RB (2021). Structure-Function Relationship of the Disintegrin Family: Sequence Signature and Integrin Interaction. Front Mol Biosci.

[B90] Kolvekar N, Bhattacharya N, Sarkar A, Chakrabarty D (2023). How snake venom disintegrins affect platelet aggregation and cancer proliferation. Toxicon.

[B91] Calvete JJ, Marcinkiewicz C, Monleón D, Esteve V, Celda B, Juárez P, Sanz L (2005). Snake venom disintegrins: evolution of structure and function. Toxicon.

[B92] Sato M, Sardana MK, Grasser WA, Garsky VM, Murray JM, Gould RJ (1990). Echistatin is a potent inhibitor of bone resorption in culture. J Cell Biol.

[B93] Knight LC, Romano JE (2005). Functional expression of bitistatin, a disintegrin with potential use in molecular imaging of thromboembolic disease. Protein Expr Purif.

[B94] Bordon K de CF, Cologna CT, Fornari-Baldo EC, Pinheiro-Júnior EL, Cerni FA, Amorim FG, Anjolette FAP, Cordeiro FA, Wiezel GA, Cardoso IA, Ferreira IG, Oliveira IS, Boldrini-França J, Pucca MB, Baldo MA, Arantes EC (2020). From Animal Poisons and Venoms to Medicines: Achievements, Challenges and Perspectives in Drug Discovery. Front Pharmacol.

[B95] Weber MA, Schiffrin EL, White WB, Mann S, Lindholm LH, Kenerson JG, Flack JM, Carter BL, Materson BJ, Ram CVS, Cohen DL, Cadet JC, Jean-Charles RR, Taler S, Kountz D, Townsend RR, Chalmers J, Ramirez AJ, Bakris GL, Wang J, Schutte AE, Bisognano JD, Touyz RM, Sica D, Harrap SB (2014). Clinical Practice Guidelines for the Management of Hypertension in the Community: A Statement by the American Society of Hypertension and the International Society of Hypertension. J Clin Hypertens (Greenwich).

[B96] Ferreira SH (1965). A bradykinin-potentiating factor (BPF) present in the venom of Bothrops jararaca. Br J Pharmacol Chemother.

[B97] Ferreira SH, Rocha e Silva M (1965). Potentiation of bradykinin and eledoisin by BPF (bradykinin potentiating factor) from Bothrops jararaca venom. Experientia.

[B98] Ferreira SH, Bartelt DC, Greene LJ (1970). Isolation of bradykinin-potentiating peptides from Bothrops jararaca venom. Biochemistry.

[B99] Ferreira SH, Greene LJ, Alabaster VA, Bakhle YS, Vane JR (1970). Activity of Various Fractions of Bradykinin Potentiating Factor against Angiotensin I Converting Enzyme. Nature.

[B100] Cushman DW, Cheung HS, Sabo EF, Ondetti MA (1977). Design of potent competitive inhibitors of angiotensin-converting enzyme. Carboxyalkanoyl and mercaptoalkanoyl amino acids. Biochemistry.

[B101] Patchett A (1984). The chemistry of enalapril. Br J Clin Pharmacol.

[B102] Topol EJ, Byzova TV, Plow EF (1999). Platelet GPIIb-IIIa blockers. Lancet.

[B103] Hartman GD, Egbertson MS, Halczenko W, Laswell WL, Duggan ME, Smith RL, Naylor AM (1992). Non-peptide fibrinogen receptor antagonists. 1. Discovery and design of exosite inhibitors. J Med Chem.

[B104] Lang SH, Manning N, Armstrong N, Misso K, Allen A, Di Nisio M, Kleijnen J (2012). Treatment with tirofiban for acute coronary syndrome (ACS): a systematic review and network analysis. Curr Med Res Opin.

[B105] Scarborough RM, Naughton MA, Teng W, Rose JW, Phillips DR, Nannizzi L, Arfsten A, Campbell AM, Charo IF (1993). Design of potent and specific integrin antagonists. Peptide antagonists with high specificity for glycoprotein IIb-IIIa. J Biol Chem.

[B106] Scarborough RM (1999). Development of eptifibatide. Am Heart J.

[B107] Tcheng JE, O’Shea JC (2002). Eptifibatide: a potent inhibitor of the platelet receptor integrin glycoprotein IIb/IIIa. Expert Opin Pharmacother.

[B108] Abbade LPF, Barraviera SRCS, Silvares MRC, Lima ABBDCO, Haddad GR, Gatti MAN, Medolago NB, Carneiro MTR, Santos LD, Ferreira RS, Barraviera B (2021). Treatment of Chronic Venous Ulcers With Heterologous Fibrin Sealant: A Phase I/II Clinical Trial. Front Immunol.

[B109] Search Results Beta ClinicalTrials.gov.

[B110] Chao BH, Jakubowski JA, Savage B, Chow EP, Marzec UM, Harker LA, Maraganore JM (1989). Agkistrodon piscivorus piscivorus platelet aggregation inhibitor: a potent inhibitor of platelet activation. Proc Natl Acad Sci.

[B111] Williams J, Rucinski B, Holt J, Niewiarowski S (1990). Elegantin and albolabrin purified peptides from viper venoms; homologies with the RGDS domain of fibrinogen and von Willebrand factor. Biochim Biophys Acta.

[B112] Musial J, Niewiarowski S, Rucinski B, Stewart GJ, Cook JJ, Williams JA, Edmunds LH (1990). Inhibition of platelet adhesion to surfaces of extracorporeal circuits by disintegrins. RGD-containing peptides from viper venoms. Circulation.

[B113] Huang TF, Sheu JR, Teng CM, Chen SW, Liu CS (1991). Triflavin, an antiplatelet Arg-Gly-Asp-containing peptide, is a specific antagonist of platelet membrane glycoprotein IIb-IIIa complex. J Biochem.

[B114] Trikha M, Rote WE, Manley PJ, Lucchesi BR, Markland FS (1994). Purification and characterization of platelet aggregation inhibitors from snake venoms. Thromb Res.

[B115] Calvete JJ, Schrader M, Raida M, McLane MA, Romero A, Niewiarowski S (1997). The disulphide bond pattern of bitistatin, a disintegrin isolated from the venom of the viper Bitis arietans. FEBS Lett.

[B116] Kang IC, Chung KH, Lee SJ, Yun Y, Moon HM, Kim DS (1998). Purification and Molecular Cloning of a Platelet Aggregation Inhibitor from the Snake (Agkistrodon Halys Brevicaudus) Venom. Thromb Res.

[B117] Wang R, Kini RM, Chung MCM (1999). Rhodocetin, a Novel Platelet Aggregation Inhibitor from the Venom of Calloselasma rhodostoma (Malayan Pit Viper): Synergistic and Noncovalent Interaction between Its Subunits. Biochemistry.

[B118] Marcinkiewicz C, Taooka Y, Yokosaki Y, Calvete JJ, Marcinkiewicz MM, Lobb RR, Niewiarowski S, Sheppard D (2000). Inhibitory Effects of MLDG-containing Heterodimeric Disintegrins Reveal Distinct Structural Requirements for Interaction of the Integrin α9β1 with VCAM-1, Tenascin-C, and Osteopontin. J Biol Chem.

[B119] Souza DHF, Iemma MRC, Ferreira LL, Faria JP, Oliva MLV, Zingali RB, Niewiarowski S, Selistre-de-Araújo HS (2000). The Disintegrin-like Domain of the Snake Venom Metalloprotease Alternagin Inhibits α2β1 Integrin-Mediated Cell Adhesion. Arch Biochem Biophys.

[B120] Gasmi A, Srairi N, Guermazi S, Dkhil H, Karoui H, El Ayeb M (2001). Amino acid structure and characterization of a heterodimeric disintegrin from Vipera lebetina venom. Biochim Biophys Acta.

[B121] Okuda D, Morita T (2001). Purification and Characterization of a New RGD/KGD-Containing Dimeric Disintegrin, Piscivostatin, from the Venom of Agkistrodon piscivorus piscivorus: The Unique Effect of Piscivostatin on Platelet Aggregation. J Biochem.

[B122] Smith JB, Theakston RDG, Coelho ALJ, Barja-Fidalgo C, Calvete JJ, Marcinkiewicz C (2002). Characterization of a monomeric disintegrin, ocellatusin, present in the venom of the Nigerian carpet viper, Echis ocellatus. FEBS Lett.

[B123] Pinto A, Angulo Y, Jiménez R, Lomonte B (2003). Isolation of bothrasperin, a disintegrin with potent platelet aggregation inhibitory activity, from the venom of the snake Bothrops asper. Rev Biol Trop.

[B124] Calvete JJ, Moreno-Murciano MP, Theakston RDG, Kisiel DG, Marcinkiewicz C (2003). Snake venom disintegrins: novel dimeric disintegrins and structural diversification by disulphide bond engineering. Biochem J.

[B125] Wang JH, Wu Y, Ren F, Lü L, Zhao BC (2004). Cloning and Characterization of Adinbitor, a Novel Disintegrin from the Snake Venom of Agkistrodon halys brevicaudus stejneger. Acta Biochim Biophys Sin (Shanghai).

[B126] Kisiel DG, Calvete JJ, Katzhendler J, Fertala A, Lazarovici P, Marcinkiewicz C (2004). Structural determinants of the selectivity of KTS-disintegrins for the α1β1 integrin. FEBS Lett.

[B127] Fernandez JH, Silva CA, Assakura MT, Camargo ACM, Serrano SMT (2005). Molecular cloning, functional expression, and molecular modeling of bothrostatin, a new highly active disintegrin from Bothrops jararaca venom. Biochem Biophys Res Commun.

[B128] Sanz L, Chen RQ, Pérez A, Hilario R, Juárez P, Marcinkiewicz C, Monleón D, Celda B, Xiong YL, Pérez-Payá E, Calvete JJ (2005). cDNA Cloning and Functional Expression of Jerdostatin, a Novel RTS-disintegrin from Trimeresurus jerdonii and a Specific Antagonist of the α1β1 Integrin. J Biol Chem.

[B129] Sánchez EE, Galán JA, Russell WK, Soto JG, Russell DH, Pérez JC (2006). Isolation and characterization of two disintegrins inhibiting ADP-induced human platelet aggregation from the venom of Crotalus scutulatus scutulatus (Mohave Rattlesnake). Toxicol Appl Pharmacol.

[B130] Thangam R, Gunasekaran P, Kaveri K, Sridevi G, Sundarraj S, Paulpandi M, Kannan S (2012). A novel disintegrin protein from Naja naja venom induces cytotoxicity and apoptosis in human cancer cell lines in vitro. Proc Biochem.

[B131] Allane D, Oussedik-Oumehdi H, Harrat Z, Seve M, Laraba-Djebari F (2018). Isolation and characterization of an anti-leishmanial disintegrin from Cerastes cerastes venom. J Biochem Mol Toxicol.

[B132] Oliveira IS de, Manzini RV, Ferreira IG, Cardoso IA, Bordon K de CF, Machado ART, Antunes LMG, Rosa JC, Arantes EC (2018). Cell migration inhibition activity of a non-RGD disintegrin from Crotalus durissus collilineatus venom. J Venom Anim Toxins incl Trop Dis.

[B133] Ameziani M, Chérifi F, Kiheli H, Saoud S, Hariti G, Kellou-Taîri S, Laraba-Djebari F (2020). Isolation and Functional Identification of an Antiplatelet RGD-Containing Disintegrin from Cerastes cerastes Venom. Protein J.

[B134] Yeh CH, Peng HC, Yih JB, Huang TF (1998). A new short chain RGD-containing disintegrin, accutin, inhibits the common pathway of human platelet aggregation. Biochim Biophys Acta.

[B135] Musial J, Niewiarowski S, Rucinski B, Stewart GJ, Cook JJ, Williams JA, Edmunds LH (1990). Inhibition of platelet adhesion to surfaces of extracorporeal circuits by disintegrins. RGD-containing peptides from viper venoms. Circulation.

[B136] Scarborough RM, Rose JW, Naughton MA, Phillips DR, Nannizzi L, Arfsten A, Campbell AM, Charo IF (1993). Characterization of the integrin specificities of disintegrins isolated from American pit viper venoms. J Biol Chem.

[B137] Vija H, Samel M, Siigur E, Aaspõllu A, Tõnismägi K, Trummal K, Subbi J, Siigur J (2009). VGD and MLD-motifs containing heterodimeric disintegrin viplebedin-2 from Vipera lebetina snake venom. Purification and cDNA cloning. Comp Biochem Physiol B Biochem Mol Biol.

[B138] McLane MA, Kowalska MA, Silver L, Shattil SJ, Niewiarowski S (1994). Interaction of disintegrins with the αIIbβ3 receptor on resting and activated human platelets. Biochem J.

[B139] Wermelinger LS, Geraldo RB, Frattani FS, Rodrigues CR, Juliano MA, Castro HC, Zingali RB (2009). Integrin inhibitors from snake venom: Exploring the relationship between the structure and activity of RGD-peptides. Arch Biochem Biophys.

[B140] Sanz L, Chen RQ, Pérez A, Hilario R, Juárez P, Marcinkiewicz C, Monleón D, Celda B, Xiong YL, Pérez-Payá E, Calvete JJ (2005). cDNA Cloning and Functional Expression of Jerdostatin, a Novel RTS-disintegrin from Trimeresurus jerdonii and a Specific Antagonist of the α1β1 Integrin. J Biol Chem.

[B141] Yeh CH, Peng HC, Huang TF (1998). Accutin, a New Disintegrin, Inhibits Angiogenesis In Vitro and In Vivo by Acting as Integrin vβ3 Antagonist and Inducing Apoptosis. Blood.

[B142] Soszka T, Knudsen KA, Beviglia L, Rossi C, Poggi A, Niewiarowski S (1991). Inhibition of murine melanoma cell-matrix adhesion and experimental metastasis by albolabrin, an RGD-containing peptide isolated from the venom of Trimeresurus albolabris. Exp Cell Res.

[B143] Dos Santos PK, Altei WF, Danilucci TM, Lino RLB, Pachane BC, Nunes ACC, Selistre-de-Araujo HS (2020). Alternagin-C (ALT-C), a disintegrin-like protein, attenuates alpha2beta1 integrin and VEGF receptor 2 signaling resulting in angiogenesis inhibition. Biochimie.

[B144] Selistre-de-Araujo HS, Cominetti MR, Terruggi CHB, Mariano-Oliveira A, De Freitas MS, Crepin M, Figueiredo CC, Morandi V (2005). Alternagin-C, a disintegrin-like protein from the venom of Bothrops alternatus, modulates alpha2ß1 integrin-mediated cell adhesion, migration and proliferation. Braz J Med Biol Res.

[B145] Moritz MNDO, Eustáquio LMS, Micocci KC, Nunes ACC, Dos Santos PK, De Castro Vieira T, Selistre-de-Araujo HS (2018). Alternagin-C binding to α2β1 integrin controls matrix metalloprotease-9 and matrix metalloprotease-2 in breast tumor cells and endothelial cells. J Venom Anim Toxins incl Trop Dis.

[B146] Beviglia L, Stewart GJ, Niewiarowski S (1995). Effect of four disintegrins on the adhesive and metastatic properties of B16F10 melanoma cells in a murine model. Oncol Res.

[B147] Juliano D, Wang Y, Marcinkiewicz C, Rosenthal LA, Stewart GJ, Niewiarowski S (1996). Disintegrin Interaction with αvβ3Integrin on Human Umbilical Vein Endothelial Cells: Expression of Ligand-Induced Binding Site on β3Subunit. Exp Cell Res.

[B148] Trikha M, De Clerck YA, Markland FS (1994). Contortrostatin, a Snake Venom Disintegrin, Inhibits β1 Integrin-mediated Human Metastatic Melanoma Cell Adhesion and Blocks Experimental Metastasis. Cancer Res.

[B149] Ramos OHP, Kauskot A, Cominetti MR, Bechyne I, Salla Pontes CL, Chareyre F, Manent J, Vassy R, Giovannini M, Legrand C, Selistre-de-Araujo HS, Crépin M, Bonnefoy A (2008). A novel alpha(v)beta (3)-blocking disintegrin containing the RGD motive, DisBa-01, inhibits bFGF-induced angiogenesis and melanoma metastasis. Clin Exp Metastasis.

[B150] McLane MA, Joerger T, Mahmoud A (2008). Disintegrins in health and disease. Front Biosci.

[B151] Pfaff M, McLane MA, Beviglia L, Niewiarowski S, Timpl R (1994). Comparison of Disintegrins with Limited Variation in the RGD Loop in Their Binding to Purified Integrins αIIbβ3, αVβ3 and α5β1 and in Cell Adhesion Inhibition. Cell Adhes Commun.

[B152] Hammouda MB, Montenegro MF, Sánchez-del-Campo L, Zakraoui O, Aloui Z, Riahi-Chebbi I, Karoui H, Rodríguez-López JN, Essafi-Benkhadir K (2016). Lebein, a Snake Venom Disintegrin, Induces Apoptosis in Human Melanoma Cells. Toxins (Basel).

[B153] Zakraoui O, Marcinkiewicz C, Aloui Z, Othman H, Grépin R, Haoues M, Essafi M, Srairi-Abid N, Gasmi A, Karoui H, Pages G, Essafi-Benkhadir K (2017). Lebein, a snake venom disintegrin, suppresses human colon cancer cells proliferation and tumor-induced angiogenesis through cell cycle arrest, apoptosis induction and inhibition of VEGF expression. Mol Carcinog.

[B154] Lucena S, Castro R, Lundin C, Hofstetter A, Alaniz A, Suntravat M, Sánchez EE (2015). Inhibition of pancreatic tumoral cells by snake venom disintegrins. Toxicon.

[B155] Tan CH, Liew JL, Navanesan S, Sim KS, Tan NH, Tan KY (2020). Cytotoxic and anticancer properties of the Malaysian mangrove pit viper (Trimeresurus purpureomaculatus) venom and its disintegrin (purpureomaculin). J Venom Anim Toxins incl Trop Dis.

[B156] Eble JA, Niland S, Dennes A, Schmidt-Hederich A, Bruckner P, Brunner G (2002). Rhodocetin antagonizes stromal tumor invasion in vitro and other α2β1 integrin-mediated cell functions. Matrix Biol.

[B157] Yeh CH, Peng HC, Yang RS, Huang TF (2001). Rhodostomin, A Snake Venom Disintegrin, Inhibits Angiogenesis Elicited by Basic Fibroblast Growth Factor and Suppresses Tumor Growth by A Selective αvβ3 Blockade of Endothelial Cells. Mol Pharmacol.

[B158] Kim DS, Jang YJ, Jeon OH, Kim DS (2007). Saxatilin, a Snake Venom Disintegrin, Suppresses TNF-α-induced Ovarian Cancer Cell Invasion. J Biochem Mol Biol.

[B159] Kim KS, Kim DS, Chung KH, Park YS (2006). Inhibition of angiogenesis and tumor progression by hydrodynamic cotransfection of angiostatin K1-3, endostatin, and saxatilin genes. Cancer Gene Ther.

[B160] Sheu JR, Huang TF (1996). Triflavin, an Arg-Gly-Asp-containing peptide, inhibits B16-F10 mouse melanoma cell adhesion to matrix proteins via direct binding to tumor cells. J Biomed Sci.

[B161] Saviola AJ, Burns PD, Mukherjee AK, Mackessy SP (2016). The disintegrin tzabcanin inhibits adhesion and migration in melanoma and lung cancer cells. Int J Biol Macromol.

